# Adipose tissue-liver cross-talk: a route to hepatic dysfunction in pregnant women with obesity

**DOI:** 10.1042/BSR20231679

**Published:** 2024-08-14

**Authors:** Diana Sousa, Carina C. Magalhães, Paulo Matafome, Susana P. Pereira

**Affiliations:** 1 Coimbra Institute for Clinical and Biomedical Research (iCBR) and Institute of Physiology, Faculty of Medicine, University of Coimbra, 3000-548 Coimbra, Portugal; 2 CIBB—Centre for Innovative Biomedicine and Biotechnology, University of Coimbra, 3004-504 Coimbra, Portugal; 3 Clinical Academic Center of Coimbra (CACC), 3004-561 Coimbra, Portugal; 4 Institute of Pharmacology and Experimental Therapeutics, Faculty of Medicine, University of Coimbra, Azinhaga de Santa Comba, 3000-548, Coimbra, Portugal; 5 Ph.D. Programme in Experimental Biology and Biomedicine (PDBEB), Institute for Interdisciplinary Research (IIIUC), University of Coimbra, Coimbra, Portugal; 6 Polytechnic University of Coimbra, Coimbra Health School, Rua 5 de Outubro—S. Martinho do Bispo, 3046-854 Coimbra, Portugal; 7 CNC-UC—Center for Neuroscience and Cell Biology, University of Coimbra,3004-504 Coimbra, Portugal; 8 CIBB—Centre for Innovative Biomedicine and Biotechnology, University of Coimbra; 3004-517 Coimbra, Portugal; 9 Laboratory of Metabolism and Exercise (LaMetEx), Research Centre in Physical Activity, Health and Leisure (CIAFEL), Laboratory of for Integrative and Translational Research in Population Health (ITR), Faculty of Sports, University of Porto, 4200-450 Porto, Portugal

**Keywords:** Gestational obesity, hepatic steatosis, Intrahepatic lipid accumulation, Maternal metabolic health, Metabolic Dysfunction-Associated Steatotic Liver Disease, Molecular bases of diseases

## Abstract

Obesity during pregnancy has been escalating, becoming a huge problem that poses consequences not only for the health of the offspring but also for the maternal well-being. Women’s adipose and hepatic tissue metabolism undergoes significant changes during the gestational period. During pregnancy, obesity is a primary instigator of steatosis, increasing the risk of non-alcholic fatty liver disease (NAFLD), now recognized under the updated nomenclature metabolic dysfunction-associated steatotic liver disease (MASLD). Pregnant women with obesity present higher levels of free fatty acids and glucose, reduction in insulin sensitivity, and adipose tissue endocrine dysregulation. Furthermore, obesity-induced modifications in clock genes and lipid-associated gene expression within adipose tissue disrupt crucial metabolic adaptations, potentially culminating in adipose tissue dysfunction. Thus, the liver experiences increased exposure to free fatty acids through the portal vein. Higher uptake of free fatty acids into the liver disrupts hepatic lipid oxidation while enhances lipogenesis, thereby predisposing to ectopic fat deposition within the liver. This review focuses on the obesity-induced changes during pregnancy in both liver and adipose tissue metabolism, elucidating how the metabolic crosstalk between these two organs can be dysregulated in pregnant women living with obesity.

## Obesity during pregnancy: a lifelong burden

Obesity is a chronic complex disease characterized by the abnormal accumulation of adipose tissue (AT), being associated with the development of other diseases such as diabetes Type 2 (T2D) and cardiovascular diseases (CVDs). Obesity can result from hereditary factors combined with nutritional cues, lifestyle, and environment [1]. Obesity is diagnosed through the body mass index (BMI, kg/square of height in meters): overweight occurs when the BMI is within 25–30 kg/m^2^, while equal or greater than 30 kg/m^2^ corresponds to obesity [1]. Nevertheless, BMI can be misleading to diagnose obesity! In turn, waist fat accumulation is associated with the risk of diabetes and CVD, regardless of BMI [2]. So, the waist-to-hip ratio (WHR) can also be used to diagnose obesity, providing a better indicator of the risk of developing obesity-associated diseases [1]. Despite having a normal BMI, an individual can be metabolically unhealthy and, consequently have a higher risk of developing obesity-associated diseases [3]. Based on this evidence, obesity can be classified into two phenotypes: metabolically healthy obesity (MHO) and metabolically unhealthy obesity (MUO) [4]. On the one hand, the MHO is characterized by insulin sensitivity, lower levels of inflammation, normoglycemia, a higher amount of subcutaneous adipose tissue (SAT), and a lower presence of visceral adipose tissue (VAT) [4]. On the other hand, individuals with MUO present insulin resistance, inflammation, hyperglycemia, dyslipidemia, and increased abdominal deposition of visceral fat mass [4]. Besides the type of adipose tissue present in each phenotype of obesity, its composition/function has also been demonstrated to be different. MHO adipose tissue is characterized by smaller hyperplasic and well irrigated adipocytes while MUO is characterized by the presence of larger hypertrophic and hypoxic adipocytes, being associated with adipose tissue inflammation, fibrosis and oxidative stress, which ultimately leads to insulin resistance and metabolic dysregulation [5].

Obesity can stem from a myriad of factors, some of which are modifiable while others are not, or from a combination of both [6]. Although obesity is not predetermined by genetic factors, genetics has a great influence on its development. Obesity caused by genetic factors can be classified as monogenic, syndromic, and polygenic obesity [1,6]. Among the modifiable factors are epigenetic alterations, lifestyle (diet and exercise), gut dysbiosis, medication, and mental health [1,6].

The incidence and prevalence of obesity have been rising worldwide since the 1970s. Nowadays, more than 2 billion adult people live with overweight, which represents almost 40% of the population worldwide, with 650 million of individuals being affected by obesity [7]. Furthermore, in 2020, more than 5 million children under the age of 5 years lived with obesity and 340 million children of the ages between 5 and 18 years presented overweight/obesity [7].

Obesity affects more women than men in almost all countries in the world [8]. Several factors can explain this sexual discrepancy, not only physiological mechanisms but also sociocultural and environmental reasons [8]. On a biological basis, the fat distribution differs between men and women, in part due to the distinct sexual hormones [8]. Furthermore, chemical, structural, and functional differences in the brain between sexes may contribute to a higher prevalence of obesity in women [8]. Regarding environmental influences, stress-related eating tends to be higher in women, and sociocultural environments can shape sex-based food preferences in which women have more tendency to eat sugar-rich food [8].

In tandem with the increase in obesity occurrence, obesity during gestation has emerged as a worldwide problem, paralleling the rise in obesity among women of reproductive age [9]. According to the World Obesity Federation, in 2014, nearly 40 million pregnant women were living with overweight or obesity [10]. The physiological metabolic adaptations occurring during uncomplicated pregnancy result in significant changes within the maternal organism, a challenge that is exacerbated in obese women.

## The challenges of pregnancy-induced metabolic alterations in women with obesity

### The metabolic shift during pregnancy

During pregnancy, women's metabolism undergoes significant challenges, including alterations in fat distribution, glucose homeostasis, and AT metabolism. Throughout the gestation period, glucose, triglycerides, and cholesterol levels increase in the mother’s organism. Glucose, as the main energy source for fetoplacental growth, is provided in high levels by the mother; triglycerides are a source of free fatty acids (FFAs) that are indispensable not only for fetal development but also for the membrane structure; cholesterol is highly demanded for embryonic and fetal development, defining the fluidity and permeability of cellular membranes [11]. In women during their reproductive age, fat accumulation predominantly occurs in the lower body (gluteofemoral area) while in men fat tends to accumulate in the upper abdominal region [12,13]. Nevertheless, fat distribution in pregnant women shifts from lower body accumulation to upper abdominal region which is more associated with VAT than with SAT [14]. Furthermore, the first phase of gestation is characterized by anabolic processes, such as hyperplasia of adipocytes and a higher rate of AT lipogenesis [14]. In mild gestation, the switch to catabolic status occurs, increasing the turnover of FFA and higher levels of lipolysis [14]. Additionally, insulin sensitivity is compromised in late pregnancy in response to the human placental growth hormone (PGH) which increases across pregnancy, affecting not only glucose metabolism but also the lipolysis rate in AT [11,14]. To accompany this impairment in insulin sensitivity, the human placental lactogen (hPL) levels increase 30 times during gestation, stimulating the maternal β-cells proliferation, mass and insulin secretion, to maintain the adequate levels of insulin and lower the threshold for glucose-stimulated insulin secretion [11,15–17]. In relation to adipokines, there are notable fluctuations in their levels that could influence insulin sensitivity. During gestation, leptin circulating levels increase, while adiponectin levels decrease, potentially contributing to reduced insulin sensitivity [15]. Although the reduction in adiponectin levels during pregnancy can contribute to the decrease in insulin sensitivity, there is also an increase in tumor necrosis factor α (TNF-α), a factor that down-regulates insulin signaling in adipocytes and skeletal muscle [16]. Additionally, fetuin-A, a cytokine released by the liver and AT, is related to insulin resistance, and its levels are increased in pregnant women [15]. Furthermore, ghrelin known as the hunger hormone, increases its expression in the human placenta during the first trimester and decreases in the final time of gestation [18]. These increased ghrelin levels further stimulate food intake and increase AT expansion due to its adipogenic role [19–21]. Moreover, the levels of the myokine irisin released from the muscle increase in pregnant women compared with non-pregnant, inducing energy expenditure by promoting the AT beiging process [18].

The fetus is incapable of gluconeogenesis, relaying on maternal glucose [18]. Throughout pregnancy, there is a gradual decline in mother’s fasting glucose levels. The reason for such reduction remains unknown, it is hypothesized that the increase in plasma volume during pregnancy and the greater utilization of glucose for energy requirements may play a role in lowering fasting glucose levels during gestation [16]. Moreover, a higher basal glucose production in the liver is noted, possibly due to the reduced insulin sensitivity [11,15,16]. Estrogen may contribute to hepatic glucose production, as rising plasma estrogen levels throughout pregnancy enhance the production of cortisol-binding globulin in the liver, thereby promoting hepatic gluconeogenesis [18,22]. Furthermore, progesterone levels also increase during pregnancy, which was been demonstrated to down-regulate the expression of insulin receptor substrate 1 (IRS-1) and impede the translocation of glucose transporter type 4 (GLUT4) in adipocytes 3T3-L1, leading to a diminished glucose uptake [23]. Consequently, during pregnancy, hepatic glucose production is enhanced while glucose uptake into adipose tissue is impaired, resulting in elevated plasmatic glucose levels that can readily enter the placenta circulation.

Pregnancy significantly impacts lipid metabolism, leading to a two-to-four-fold increase of triglycerides levels and a rise of 25% to 50% of total cholesterol levels [16]. During the anabolic state, observed in humans and rats, the hydrolyzation of very low-density lipoprotein (VLDL) and triglyceride-rich chylomicrons is higher due to the increase in lipoprotein lipase (LPL) activity. Consequently, the uptake of non-esterified fatty acids (NEFA)/ glycerol by AT rises, promoting fat accumulation [11,24]. During late gestation, there is a reduction in suppression of glycerol turnover, indicating higher rates of lipolysis, which is caused by insulin resistance development during pregnancy [16]. Furthermore, LPL activity decreases, impairing fat deposition in the AT, while hormone-sensitive lipase (HSL) mRNA and activity increase, releasing FFA [11,25]. In this phase, NEFA and glycerol are converted into acetyl-CoA and glycerol-3-phosphate, respectively, to become an energy source for the growing fetus [11]. Genes associated with lipid metabolism in the AT are regulated by clock genes, namely Basic helix-loop-helix ARNT-like protein 1 (Bmal1), period circadian protein homolog 1 (Per1), period circadian protein homolog 2 (Per2), Cryptochrome Circadian Regulator 1 (Cry1), Cryptochrome Circadian Regulator 2 (Cry2), and Nuclear Receptor subfamily 1 group D member 1 (Rev-erbα). Although the expression of these clock genes in gonadal AT from pregnant mice rhythmicity is maintained robustly, lipogenic and lipolytic genes do not follow this pattern [26]. Interestingly, peroxisome proliferator activated receptor γ (PPARγ) which is regulated by the Per2 clock gene, maintains its rhythmicity throughout gestation [26]. Bmal1 is known to regulate the HSL and adipose triglyceride lipase (ATGL) genes. However, these genes lost their rhythmicity during pregnancy, indicating that the expression of these genes is not coupled to these clock genes [26].

Pregnancy triggers metabolic changes akin to those induced by obesity, including insulin resistance and shifts in adipokine levels and lipid metabolism. Unveiling the ramifications of obesity during pregnancy is crucial for comprehending potential deteriorations in maternal metabolism throughout gestation and their lasting effects on long-term health.

### Does obesity during pregnancy have enduring impacts across a lifetime?

Obesity *per se* induces metabolic changes but pregnant women living with obesity cope with more dramatic alterations. However, interventions to counteract obesity during pregnancy, such as bariatric surgery and anti-obese drugs are not used due to their risk to the offspring development [27]. Fat deposition in the abdominal area is more accentuated when pregnant women have obesity, contributing to higher levels of FFA in the circulation, leading to lipotoxicity and, therefore, to inflammation and endothelial dysfunction [28]. Obesity is highly associated with insulin resistance. The insulin plasma levels during pregnancy are similar between women with and without obesity [29]. However, obesity may further decrease insulin sensitivity, which impairs glucose uptake and elevate plasma FFA levels. The anorexigenic adipokine leptin present higher levels in non-pregnant individuals with obesity, which further contributes to leptin resistance characterized by the reduction of satiety and catabolic metabolism, leading to weight gain [30,31]. Leptin levels are also increased in pregnant women with obesity compared with lean pregnant women, contributing to leptin resistance during pregnancy [14]. Thus, obesity and pregnancy contribute to increased leptin plasma levels. Furthermore, adiponectin levels also have the same pattern in obesity and pregnancy, decreasing in both. Adiponectin reduction is more abrupted in pregnant women with obesity compared with normal weight pregnant women [32]. In pregnant women with a BMI greater than 30 kg/m^2^, the reduction in adiponectin levels was found to be caused by an alteration in the methylation of the adiponectin gene, diminishing its expression [14]. Therefore, pregnancy-induced changes in leptin and adiponectin signaling are accentuated in obesogenic conditions, which can lead to fat accretion and AT dysfunction [33,34].

Pregnant women with overweight and obesity have a higher predisposition to preterm delivery and miscarriage [27]. Moreover, in the postpartum phase, women with obesity have lower milk production and a higher risk of metabolic syndrome, since they are more prone to excessive gestation weight gain [27]. Furthermore, women with obesity are more susceptible to pregnancy disorders such as preeclampsia and gestational diabetes mellitus (GDM) [35]. GDM is the most common disease that occurs during pregnancy affecting 7–10% of pregnant women worldwide [36]. Women who suffer from GDM are 7 times more prone to the development of T2D within 10 years [15]. As happens in T2D, AT endocrine function changes with GDM. For instance, as happens in obesity, GDM is followed by lower adiponectin and higher leptin levels [15]. Other adipokines were described to be altered during gestation in women with obesity and GDM, such as chemerin, omentin and fetuin-A. Chemerin is involved in adipocyte development and presents higher levels in both pregnant women with obesity and GDM compared with healthy weight pregnant women [15]. Regarding insulin-sensitizing adipokine omentin, it was shown to be reduced during pregnancy in obesity and GDM conditions, while fetuin-A levels are increased during GDM compared with normal weight pregnancy [15].

The adaptation of pancreatic β-cells is essential for maintaining glucose homeostasis and plays a crucial role during pregnancy [17]. However, in cases of maternal obesity, these adaptation mechanisms can become compromised, leading to an increased susceptibility to developing hyperglycemia and GDM [17]. Several studies reported greater β-cells mass, but insulin secretion disruption in pregnant mice and rats fed a high-fat diet [37,38]. Furthermore, in pregnant rodent models fed with a high-fat high-sugar diet it was also observed impaired β-cell proliferation, glucose intolerance and a decrease in the expression of hPL and prolactin-related genes [39,40]. Besides exacerbating the hormonal alterations induced by pregnancy and affecting insulin release, obesity can also increase pro-inflammatory cytokines characteristic of the pro-inflammatory profile of pregnancy, inducing the dedifferentiation of β-cell and decreasing the insulin secretory activity [17,41].

The impact of obesity during pregnancy in women’s organisms is poorly studied, leaving many questions unanswered, namely whether the systemic metabolic changes are caused solely by obesity or by synergy with pregnancy-induced alterations. The adaptations of the AT metabolism during this phase are necessary for the proper nutrient supply to the fetus. However, the impact of obesity on such adaptations is still unknown. In fact, several adipokines levels change during pregnancy in unhealthy conditions, demonstrating that changes occurring in the AT may have a critical role in the metabolic consequences of obesity during pregnancy, maybe not only for the mother but also for the offspring’s future health.

## Adipose tissue-liver cross-talk dysfunction during obesity

### Adipose tissue alterations during obesity

As has been discussed above, the remodeling of AT is affected by both obesity and pregnancy, altering not only AT metabolism but also its endocrine function and inflammatory environment. During pregnancy in women with obesity, the AT presents an inflammatory environment, insulin resistance, and endoplasmic reticulum (ER) stress which causes an imbalance between pro-inflammatory and anti-inflammatory adipokines contributing to gestational problems [42]. For instance, the SAT adipocytes of pregnant women with obesity have the double size of adipocytes of SAT from healthy pregnant women [14]. Furthermore, insulin binding in SAT adipocytes naturally decreases by 50% during pregnancy and this reduction in insulin binding is more accentuated in women living with obesity, leading to a higher lipolytic activity due to the lower suppression of lipolysis by insulin [14]. Obesity-induced alterations in AT during pregnancy also change the gene expression of several genes involved in fatty acid uptake and adipogenesis in both omental and SAT. The expression of LPL, a protein responsible for breaking the triglycerides from lipoprotein into FFA, and its transcriptional regulator PPARγ mRNA levels are also reduced in omental AT from pregnant women with obesity compared with lean pregnant women on the day of partum [43]. The omental AT of these women secrets more TNF-α, interleukin-1β (IL-1β), and leptin compared with non-obese pregnant women. Interestingly, in adipocytes extracted from pregnant women on partum day, TNF-α and IL-1β also reduce the mRNA levels of Acyl-CoA synthetase-1 (ACSL1), fatty acid transport protein 2 (FAPT2) and ATGL [43], proteins involved in the conversion of long-chain FFA into short-chain FFA, in the uptake of FFA from extracellular regions into adipocytes and the breakdown of triglycerides stored in the AT, respectively [43,44]. In *ex vivo* conditions, it was observed that exposure of omental AT from pregnant women to leptin and IL-1β reduced fatty acid transport protein 6 (FATP6) and peroxisome proliferator activated receptor δ (PPARδ) mRNA, compared with non-obese women [43]. However, other transcription factors such as liver X receptor α (LXRa), peroxisome proliferator activated receptor α (PPARα), retinoid X receptor α (RXRα) and sterol regulatory element-binding protein 1 (SREBP1c) that are reduced in omental AT during obese pregnancy independently of TNF-α, IL-1β, and leptin action [43]. In addition, decreased levels of α-1,3-mannosyl-glycoprotein 2-β-N-acetylglucosaminyltransferase (MGAT1) and α-1,6-mannosyl-glycoprotein 2-β-N-acetylglucosaminyltransferase (MGAT2) were also observed in omental AT from pregnant women with obesity [43], suggesting that triglyceride synthesis is impaired in such conditions.

On SAT, a reduction in fatty acid-binding protein (FABP), diacylglycerol O-acyltransferase 1 (DGAT1), and HSL mRNA levels were observed in pregnant women with obesity on partum day, indicating that both lipolysis and lipogenesis are reduced [43]. AT dysfunction is caused by obesity *per se*; however, the metabolic challenges promoted by pregnancy also alter the AT gene profile. For instance, the expression of PPARγ is reduced on SAT of pregnant women with obesity compared with non-pregnant women with similar BMI [14]. Thus, pregnancy with obesity changes lipid metabolism in AT, affecting not only the AT capacity for storage but also the ability of lipid oxidation. Furthermore, it was shown that women with obesity during pregnancy have a lower capacity to oxidate lipids compared with healthy women, affecting the energy required for gestation during the catabolic phase [45]. Hence, since energy storage mechanisms are reduced in obesity during pregnancy, it can lead to the decrease of lipolytic gene expression, attempting to balance energy expenditure and storage at the end of gestation. Therefore, more studies are needed to understand if the decrease observed in the lipolytic genes in the AT from pregnant women with obesity at the partum day is a response to the lower adipogenic activity or if it is caused by the incapacity of AT to readjust the catabolic processes for the amount of nutrients present in the obesogenic condition.

**Figure 1:**
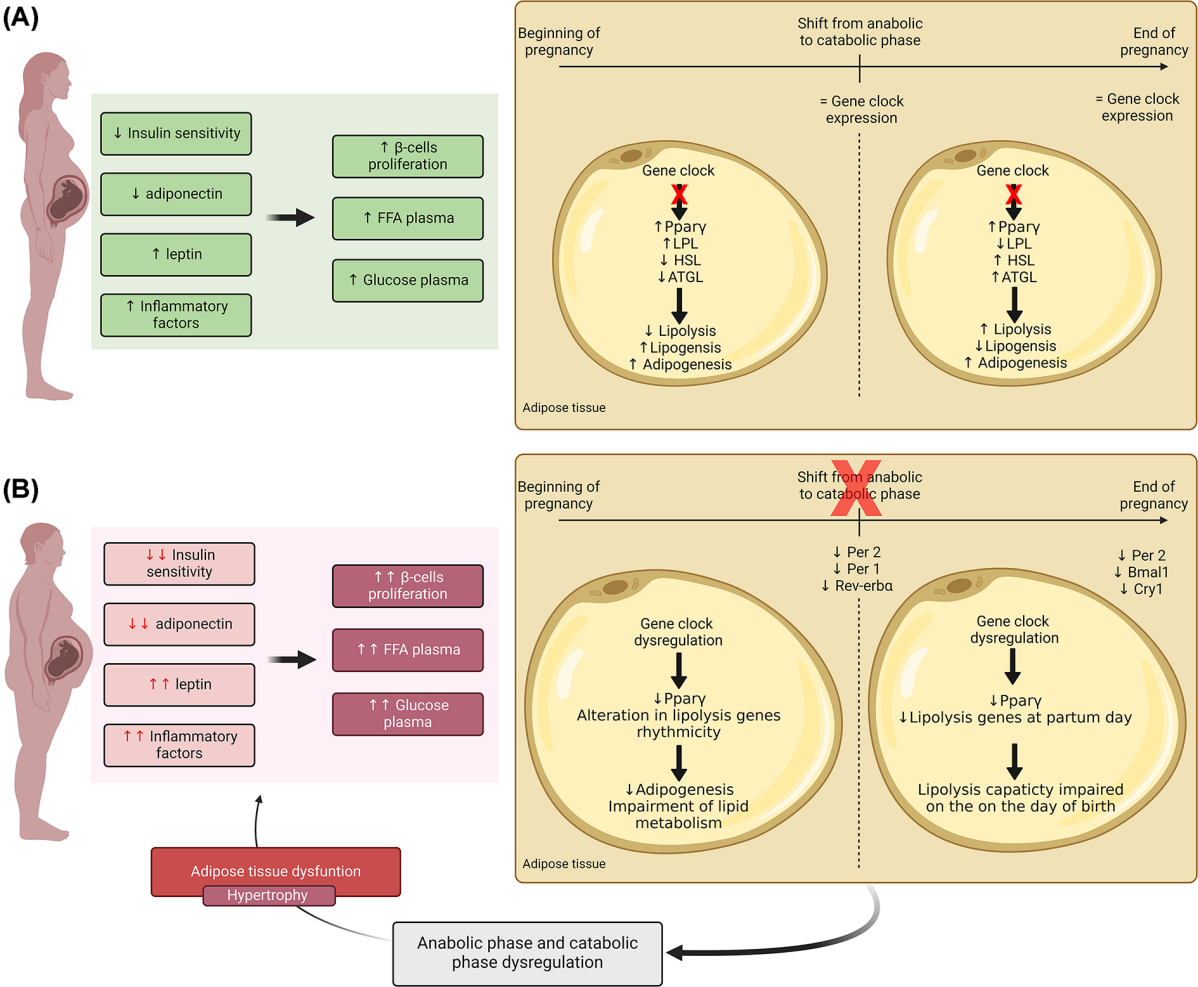
Pregnancy-induced alterations in adipose tissue: insights in maternal metabolism (**A**) During the gestation period, changes in maternal inflammatory factors, hormone levels and insulin sensitivity leads to an increase in the plasma levels of FFA and glucose. In adipose tissue, pregnancy alters in the expression of lipid metabolism-associated genes without changing the clock gene expression. In the first period of gestation, LPL, HSL and ATGL expression is altered, promoting anabolic mechanisms while the lipolysis rate is suppressed. At mid-gestation, a shift from an anabolic to a catabolic state occurs, in which LPL expression is reduced and HSL and ATGL expression increased. The expression of PPARγ is up-regulated throughout pregnancy, allowing adipogenesis. (**B**) Obesity exacerbates the alterations induced by pregnancy in insulin sensitivity, adiponectin, leptin, and inflammatory factors plasma levels, promoting a more accentuated increase in FFA and glucose plasma levels. On adipose tissue, clock genes expression changes with obesity during pregnancy. Furthermore, PPARγ expression reduces during gestation, impairing adipogenic capacity in adipose tissue. Dysregulation of lipid metabolism-associated genes in obesity leads to the loss of the shift in metabolic state induced by pregnancy at mid-gestation. ATGL, adipose triglyceride lipase; Bmal1, Basic helix-loop-helix ARNT-like protein 1; Cry 1, Cryptochrome Circadian Regulator 1; FFA, free fatty acid; HSL, hormone-sensitive lipase; LPL, lipoprotein lipase; Per1, Period Circadian Protein Homolog 1; Per2, Period Circadian Protein Homolog 2; PPARγ, peroxisome proliferator activated receptor γ.

Obesity also impacts clock genes expression in AT. Visceral adipocytes isolated from women with obesity showed that during 24 h, Bmal1 expression changes in the first 12 h in visceral adipocytes compared with lean women, while Rev-reb and Cry2 expression is up-regulated during the 24 h in visceral and subcutaneous adipocytes [46]. Rev-reb suppresses antiadipogenic genes [46], thus, with obesity, adipogenic genes may be up-regulated due to Rev-reb overexpression. Elaine Vieira et al. showed that the clock gene expression alterations in the AT are independent of the systemic clock [46]. Furthermore, it was observed that the expression is up-regulated during 24 h in adipocytes isolated from women with obesity compared with healthy ones. Nevertheless, its target PPARγ is only up-regulated at the 0 h and at the 24 h [46]. Thus, obesity alters AT clock genes that regulate lipid metabolism-associated genes. During pregnancy, obesity contributes to changes in gene clock expression. In an obesity animal model induced by a hypercaloric diet, retroperitoneal AT from pregnant rat showed at the mid-gestation lower levels of Per1 and Rev-erbα expression, in late pregnancy a reduction of Bmal1 and Cry1 and throughout the gestation period, it was observed a down-regulation of the gene Per2 (Figure 1) [46,47]. In addition, the peak of gene expression is altered with obesity during pregnancy. Thus, while during a healthy pregnancy clock gene expression in the AT presents rhythmicity, under obesogenic conditions there is dysregulation of this clock. Interestingly, Per1, Per2, and Cry2 expression is unchanged in obesity but alters in pregnant obese rats [47]. Regarding genes associated with lipid metabolism, it was already discussed that rhythmicity is lost during pregnancy. In AT from obese rats, it was observed that pregnancy restores rhythmicity in lipolytic genes, namely pparα, pparδ and peroxisome proliferator-activated receptor-γ coactivator α (Pgc1α), in mid-gestation [47]. Thus, it suggests that during the anabolic phase, the expression of lipolytic genes in AT are dysregulated (Figure 1). On the contrary, the rhythmicity of pparγ expression in AT during lean pregnancy maintained over gestation is lost in obesogenic conditions in mid-gestation (Figure 1) [47]. In conclusion, during obese pregnancy pparγ is down-regulated which may be a consequence of the reduction of its gene clock regulator Per2 expression, and, consequently, the AT may lose its adipogenic capacity. Regarding lipolysis, ATGL reduction in VAT at the partum day may be caused by the reduction of Bmal1 expression and by the increase in inflammatory factors, impairing the first step of lipolysis and the released of the required energy from the maternal AT. Therefore, the disruption of maternal AT metabolism caused by obesity may lead to AT dysfunction and lipotoxicity exacerbating insulin resistance.

Regarding the endocrine function of AT, insulin resistance plays an important role in the regulation of adiponectin levels in obesity and pregnancy. As already mentioned, pregnant women living with obesity present lower adiponectin levels compared with healthy pregnant women. The ubiquitination of adiponectin in obese pregnant mice is higher in the gonadal AT [48]. In 3T3-L1 cells, a murine preadipocyte cell line, insulin was found to increase adiponectin levels by decreasing the rate of adiponectin ubiquitination [48]. Furthermore, the activation of endoplasmic reticulum (ER) stress pathways and inflammation inhibit the insulin effect on adiponectin ubiquitination in 3T3-L1 cells [48]. Therefore, during pregnancy, obesity-induced insulin resistance decreases adiponectin levels, but also the pro-inflammatory environment and ER stress by inhibiting insulin action in preventing adiponectin ubiquitination [48].

Overall, in pregnancy with obesity, both SAT and VAT undergo metabolic alteration that contributes to lipotoxicity by increasing FFA levels in the plasma. Obesity induces the secretion of inflammatory factors, increases leptin plasmatic levels, dysregulates the clock genes from AT, and exacerbates insulin resistance in pregnant women. These changes reduce the expression of adipogenic-associated genes contributing to AT hypertrophy and hypoxia, and to the reduction of energy storage capacity.

### Portal vein, the route to liver alterations during obesity

#### The hepatic impact of obesity during pregnancy: exploring the portal vein theory

**Figure 2:**
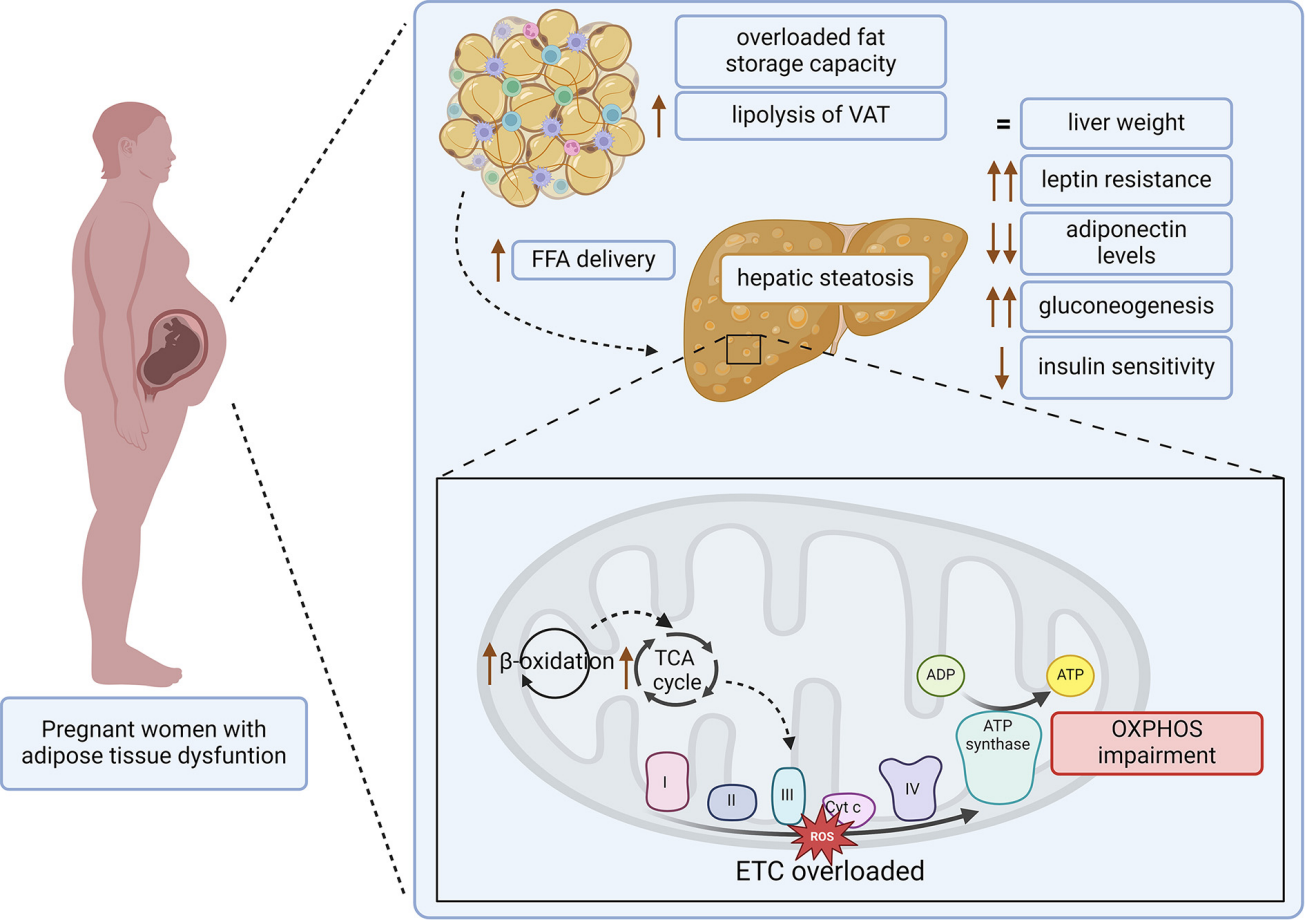
Pathophysiological mechanisms of obesity-induced hepatic and metabolic dysfunction during pregnancy During pregnancy with obesity, fat storage capacity is exceeded and the lipolysis rate in subcutaneous adipose tissue is elevated, inducing an increase in VAT and ectopic fat accumulation, namely in the liver. The excessive hepatic FFAs uptake stimulates β-oxidation and TCA cycle overload, increasing reactive oxygen species (ROS) production and impaired OXPHOS. Additionally, insulin and leptin resistance are exacerbated in pregnant women with obesity, while the reduction of adiponectin levels is accentuated. These hormonal alterations contribute to glucose intolerance and dysregulation of gluconeogenesis. Moreover, the pregnancy-induced hepatomegaly that occurs during a healthy pregnancy is harmed. BMI, body mass index; ETC, electron transport chain; FFA, free fatty acid; OXPHOS, oxidative phosphorylation; TCA cycle, tricarboxylic acid cycle; VAT, visceral adipose tissue.

The ‘portal hypothesis’ posits that in obese individuals, the liver faces direct exposure to elevated levels of FFA and cytokines released from visceral fat deposits into the portal vein. This increased exposure significantly increases the notably perilous risk associated with visceral fat accumulation significantly contributing to the onset of hepatic insulin resistance, the progression toward T2D, and ultimately promoting MASLD and/or liver steatosis (Figure 2).

As previously described, during pregnancy, the high energy demand required to sustain the fetus’s development promotes a strong metabolic adaptation. Pregnancy is characterized by an initial increase in lipid synthesis and fat stores [14]. However, the AT plasticity is limited. When the ability of SAT to store fatty acids is overloaded, it leads to visceral and ectopic accumulation and subsequent impairment of the insulin signaling pathway [15,49]. In women with obesity, visceral fat accumulation begins earlier, as they have more subcutaneous fat accretion at periconception [50]. Due to the high rate of lipolysis of VAT, the fatty acids are directly delivered to the liver via the portal vein [51,52]. In hepatocytes, the excessive FFA uptake stimulates catabolic metabolism [53]. The increased β-oxidation rate and subsequent production of reducing equivalents and substrates resulting from tricarboxylic acid cycle (TCA cycle) overload the electron transport chain, increasing reactive oxygen species (ROS) production, which leads to oxidative phosphorylation (OXPHOS) impairment (Figure 2) [53]. In agreement, in a recent study in an obese pregnant sheep model at late gestation, it was observed an increase in the liver mitochondrial respiratory chain complex I (NADH dehydrogenase) and complex-IV (cytochrome *c* oxidase) activities, probably due to the high β-oxidation rate [48]. To counteract a possible increase in ROS production, an increase in superoxidase dismutase (SOD) activity was observed in the liver of obese pregnant sheep [53]. However, the levels and activity of catalase and glutathione peroxidase (Gpx) activity significantly decreased, justifying the hepatic increase of malondialdehyde (MDA) levels, an indicator of lipid peroxidation and increased oxidative stress. Moreover, it was observed a decrease in the reduced nicotinamide adenine dinucleotide (NADH) levels, and a consequent increase in the oxidized nicotinamide adenine dinucleotide (NAD+)/NADH ratio levels, as well as a decrease in protein expression of mitochondrial complex subunits (Ndufs8, SDHA, SDHB, UQCRFS1) in the hepatic tissue from obese pregnant sheep [53]. The authors suggest that these alterations occur because of OXPHOS impairment caused by increased hepatic oxidative stress in pregnant sheep with obesity [53]. Furthermore, high nutrient intake induces a shift in liver metabolism, enhancing hepatic anabolic pathways [53]. Therefore, the combination of increased FFA uptake and lipogenesis with a decrease in fatty acid oxidation and synthesis of VLDL in the liver, can contribute to liver insulin resistance and hepatic steatosis [51,52]. Indeed, it has been reported that women with obesity are more predisposed to developing fatty liver disease during pregnancy [51,54,55]. Given the liver’s high susceptibility to fat accumulation, it is imperative to investigate hepatic alterations linked to maternal obesity. Such exploration is crucial for preventing and mitigating the health implications for both mothers and their offspring.

An important adaptive mechanism of the liver is gestational hepatomegaly, the increase in liver size during pregnancy (Figure 2) [56]. In mice, at mid-gestation (12 days), it was observed that the increase in liver weight was disproportional to maternal body weight; however, at the 18-day, the liver-body weight ratio became similar to non-pregnant mice, suggesting the normalization of liver size in late pregnancy [57]. This is a process characterized by both hepatocyte hyperplasia and hypertrophy [56]. Liver growth appears to be positively related to maternal body weight gain and the number of fetuses [57]. Furthermore, in the CD-1 mouse model, the maternal liver enlargement during pregnancy involves an hepatic up-regulation of interleukin 6 (IL-6) and TNF-α, nuclear factor kB (NF-kB), c-Jun, and IL1-β and activation of the signal transducer and activator of transcription 3 (STAT3), β-catenin and epidermal growth factor receptor (EGFR), revealing that some mechanisms are common between pregnancy-induced hepatomegaly and liver regeneration [56]. However, liver steatosis has been reported to affect liver regeneration [58]. Although during healthy pregnancies in humans [59] and rodents [60,61] pregnancy-induced hepatomegaly occurs. However, at late gestation, Grilo et al. did not observe alterations in liver weight in a maternal obese sheep model [53]. The authors verified mitigation in the increase of liver size to 14% in obese pregnant sheep at late gestation. Moreover, the increased activity of alanine aminotransferase (ALT) and alkaline phosphatase (ALP) observed, suggests higher liver damage [53]. Thus, the pregnancy-induced liver growth process may be harmed in mothers with obesity.

As mentioned in the section above, a normal pregnancy generally follows a pattern of hyperinsulinemia and a progressive decrease in insulin sensitivity [62], which means that at early gestation, pregnant women are more insulin sensitive than at late pregnancy. The insulin sensitivity pattern during pregnancy appears to be in agreement with the phosphorylation pattern of IRS-1 in pregnant female Wistar rats [62]. In fact, it was observed that IRS-1 phosphorylation was higher in AT during early pregnancy than at the end of this period, which is in line with decreased insulin sensitivity throughout pregnancy [62]. Nevertheless, González et al. observed that on day 10, pregnant Wistar rats peaked at the maximum phosphorylation of IRS-1 in AT, while the minimum phosphorylation of IRS-1 was observed in the liver, indicating that insulin signalling reaches its lowest at the hepatic level at mid-gestation. The authors assumed that day 10 of gestation may be the inflection point that divides the early from the late pregnancy in terms of insulin sensitivity for this animal model [62]. Furthermore, IRS-1 phosphorylation and subsequent insulin sensitivity appear to be positively associated with plasma levels of estradiol and progesterone [62]. Steroid hormone production can be affected by the dysregulation of cholesterol production in the liver observed in women with obesity and by lower mitochondrial cholesterol concentrations in the placenta of obese pregnancy [51,63]. Accordingly, it was reported that women with BMI >30 kg/m^2^ have lower plasma and placental levels of estradiol and progesterone [63] possibly due to higher insulin resistance than lean pregnant women [14]. As a result, the decreased insulin’s ability to down-regulate adipocyte lipolysis in pregnant women with obesity leads to an increase in FFA delivery to the liver and VLDL synthesis in the liver [14]. Furthermore, during normal late pregnancy, a decrease in insulin sensitivity, an increase in insulin production, and an increase in liver glucose production contribute to maintaining glucose homeostasis [64]. Since the human fetus is highly dependent on the mother’s blood supply of glucose, a decrease in fasting glucose was observed in lean women. However, pregnant women with obesity presented fasting hyperglycemia [64]. In fact, Catalano and colleagues observed that women with obesity and GDM had a lower suppression of endogenous glucose production (gluconeogenesis and glycogenesis) during insulin infusion, predisposing these women to a higher risk of developing T2D later in later life [65,66].

The alterations in the endocrine function of AT during pregnancy are closely related to the adjustments observed in maternal hepatic function (Figure 2). Leptin and adiponectin reach the liver via the portal vein, modulating maternal hepatic lipid and glucose metabolism. A healthy pregnancy is characterized by a state of central leptin resistance, which allows an increase in fat energy stores (anabolic metabolism) and increased food intake to guarantee that the fetus receives sufficient nutrition [67]. More recently, it has been shown that, in rats, leptin resistance can not only be central but also peripheral. Both liver and SAT demonstrated a biphasic response to hyperleptinemia that occurs during pregnancy [68]. At mid-gestation, a decrease in leptin receptor b (LepRb) mRNA and an increase in protein expression of suppressor of cytokine signaling 3 (SOCS3), an inhibitor of LepRb-STAT3 signaling, was observed in both peripheral organs. Additionally, the activity and protein content of the main molecules involved in *de novo* lipid synthesis, such as SREBP-1 and fatty acid synthase (FAS) increased while proteins involved in β-oxidation such as PPARα and carnitine palmitoyltransferase 1 (CPT-1) decreased in the liver [68]. However, at the end of gestation, there was a recovery of the response to leptin, favoring catabolic metabolism [68]. In maternal obesity conditions, the maternal serum leptin levels are even higher than in lean pregnancy since leptin release is proportional to the amount of AT (Figure 1) [69]. Although the placenta has also the ability to produce leptin, this increase appears to be solely due to the high adiposity observed in women with obesity [69]. Furthermore, decreased expression of the placental leptin receptor was observed in pregnant women with obesity, indicating that hyperleptinemia associated with maternal obesity may induce placental leptin resistance, which appears to negatively impact placental amino acid transport [69]. In contrast, the BMI of pregnant women is negatively correlated with plasma adiponectin levels [70]. Interestingly, low plasma levels of adiponectin have been associated with metabolic dysfunction-associated steatotic liver disease (MASLD) in individuals with obesity [71,72]. A recent study reported that although high-fat diet-fed mice presented higher steatosis score, both low-fat and high-fat diet-fed pregnant adiponectin knockout (KO) mice developed hepatic steatosis in the third week of pregnancy [73]. Pregnant adiponectin KO mice also showed hyperglycemia, impaired glucose tolerance, and increased hepatic triacylglycerol, being more pronounced in animals fed with the high-fat diet. However, adiponectin administration by tail-vein injection improved glucose tolerance and decreased the expression of hepatic lipogenic genes such as SREBP-1 that were elevated in adiponectin KO mice [73]. Thus, in the liver, adiponectin suppresses gluconeogenesis, glucose output, and lipid accumulation [71,73]. However, it is important to note that although adiponectin administration decreased hepatic triacylglycerol, it was not sufficient to improve glucose tolerance in pregnant mice fed a high-fat diet [73]. In conclusion, during pregnancy, women with obesity may be more prone to developed steatosis due to the higher lipogenesis activity but also due to the greater FFA efflux from AT to the liver via the portal vein, underscoring the importance of conducting thorough assessments of the hepatic function within this population (Figure 2).

## Conclusion and future perspectives

Obesity impairs pregnancy-induced adaptations in lipid metabolism. Both obesity and pregnancy decrease insulin sensitivity and increase the levels of leptin and inflammatory factor, promoting an increase in cholesterol, FFA, triglycerides, and glucose plasma levels. The shift from subcutaneous lipid accumulation to visceral fat deposition in pregnancy and obesity may be behind these alterations. Pregnancy with obesity is associated with further changes in metabolism, in which pregnant women with obesity present higher levels of leptin and glucose as well as higher adipocytes size. Nevertheless, the absence of studies comparing metabolic parameters including all clinical conditions (healthy non pregnant, healthy pregnant, non-pregnant with obesity, and pregnant with obesity) does not allow us to conclude whether there is a synergistic effect between pregnancy and obesity. Obesity itself is highly associated with AT dysfunction. During pregnancy, AT plays a crucial role in energy management, being more adipogenic/lipogenic in the first phases of gestation, shifting to a catabolic state at mid-gestation. With obesity, the anabolic phase can be compromised due to the lower levels of PPARγ in AT during pregnancy, which contributes to lipotoxicity. Interestingly, at the last day of gestation, lipolysis may be impaired in pregnant women with obesity. However, the reason for this is still unknown. Since AT also presents lower energy storage capacity and plasma FFA levels are higher in obesogenic conditions, it is possible that in the attempt to balance the FFA levels, the gene expression of lipolytic genes is reduced. As happens in nonpregnant women with obesity, dysregulation of lipid metabolism and insulin sensitivity contributes to hepatic steatosis due to increased plasma FFA deposition as ectopic fat through the portal vein. Pregnant women living with obesity may be more susceptible to the development of liver steatosis due to the risk associated with obesity being potentiated by the natural metabolic adaptations of pregnancy. Furthermore, β-oxidation at the hepatic level is reduced in pregnant women with obesity, and combined with decreased VLDL formation, fat accumulation in the liver may increase. Dysregulation of adipokines levels also contributes to steatosis, since the decrease in adiponectin levels impairs insulin signaling in the liver and, therefore, there is a reduction in the inhibitory effect in lipid accumulation. Steatosis is the first step of MASLD. Hence, obesity increases the risk of diabetes and the development of MASLD. In pregnant women, obesity may predispose to metabolic diseases development since the metabolic changes induced by pregnancy overlap/extend the ones observed in obesogenic conditions, aggravating the metabolic maternal profile. In this review, we observed the lack of information regarding the impact of obesity on the maternal metabolic adaptations during the gestation period. Given that obesity prevalence continues to increase in the last years, more studies are required to understand how obesity can be managed during pregnancy to improve both maternal and offspring metabolic state in order to decrease the susceptibility to metabolic diseases development.

## Abbreviations

ALT: alanine aminotransferase
ALP: alkaline phosphatase
AT: adipose tissue
BMI: body mass index
CPT-1: carnitine palmitoyltransferase 1
CVD: cardiovascular disease
EGFR: epidermal growth factor receptor
FAS: fatty acid synthase
GLUT4: glucose transporter type 4
HSL: hormone-sensitive lipase
IL-6: interleukin 6
IRS-1: insulin receptor substrate 1
LPL: lipoprotein lipase
MASLD: metabolic dysfunction-associated steatotic liver disease
MDA: malondialdehyde
MHO: metabolically healthy obesity
MUO: metabolically unhealthy obesity
NAFLD: non-alcholic fatty liver disease
NEFA: non-esterified fatty acid
NF-kB: nuclear factor kB
OXPHOS: oxidative phosphorylation
ROS: reactive oxygen species
SAT: subcutaneous adipose tissue
SOCS3: suppressor of cytokine signaling 3
SOD: superoxidase dismutase
STAT3: signal transducer and activator of transcription 3
T2D: Type 2 diabetes
TCA cycle: tricarboxylic acid cycle
TNF-α: tumor necrosis factor α
VAT: visceral adipose tissue
VLDL: very low-density lipoprotein
WHR: waist-to-hip ratio
